# The interaction of hydrogen with the {010} surfaces of Mg and Fe olivine as models for interstellar dust grains: a density functional theory study

**DOI:** 10.1098/rsta.2011.0592

**Published:** 2013-07-13

**Authors:** C. A. Downing, B. Ahmady, C. R. A. Catlow, N. H. de Leeuw

**Affiliations:** Department of Chemistry, University College London, 20 Gordon Street, London WC1H 0AH, UK

**Keywords:** olivine, surfaces, hydrogen, adsorption, density functional theory

## Abstract

There is no consensus as yet to account for the significant presence of water on the terrestrial planets, but suggested sources include direct hydrogen adsorption from the parent molecular cloud after the planets’ formation, and delivery of hydrous material via comets or asteroids external to the zone of the terrestrial planets. Alternatively, a more recent idea is that water may have directly adsorbed onto the interstellar dust grains involved in planetary formation. In this work, we use electronic structure calculations based on the density functional theory to investigate and compare the bulk and {010} surface structures of the magnesium and iron end-members of the silicate mineral olivine, namely forsterite and fayalite, respectively. We also report our results on the adsorption of atomic hydrogen at the mineral surfaces, where our calculations show that there is no activation barrier to the adsorption of atomic hydrogen at these surfaces. Furthermore, different surface sites activate the atom to form either adsorbed hydride or proton species in the form of hydroxy groups on the same surface, which indicates that these mineral surfaces may have acted as catalytic sites in the immobilization and reaction of hydrogen atoms to form dihydrogen gas or water molecules.

## 1. Introduction

The formation of stars and planets occurs via a complex and staged process spread over millions of years, which is not yet fully understood. What is known is that the process begins within relatively dense concentrations of interstellar gas and dust known as molecular clouds (MCs). The extremely low temperatures of the clouds—studies have found that temperatures range between 10 and 20 K—favour the formation of molecules, as opposed to ions.

MCs are considered to act as ‘stellar nurseries’ as their denser core areas collapse under gravity to form clusters of protostars—a type of young stellar object (YSO), or early-stage star—which develop circumstellar discs in orbital motion around themselves, accreting material gradually from the parent MC. These accretion discs are largely made up of gases and dust particles, which comprise carbonaceous and siliceous materials. Astronomical observations, for example, have shown that dust clouds around YSOs consist of Mg-rich olivines, pyroxenes and other refractory minerals with radii less than 1 μm. Several authors have suggested that these refractory minerals could coalesce by means of low-velocity impacts that would create low-density, irregularly shaped fractal structures [1–3].

In conjunction with observations by Eisner [4], who showed the presence of water vapour and atomic hydrogen in the dust and gas around the YSO MWC80 at 1 AU (astronomical unit, corresponding to the mean distance between the Earth and the Sun—equal to exactly 149 597 870 700 m), we can postulate a scenario whereby water is incorporated into the Earth's building material by means of gas–solid interactions. Although theoretical estimates of the lifetime of gas in the accretion disc vary greatly from 0.1 to 100 My [5], even the lowest estimate should allow sufficient time for adsorption. If strong bonds could be developed during adsorption, the products would be resistant to desorption even at high temperatures [4,6–13].

In addition to the adsorption of water molecules from the vapour phase, adsorption of hydrogen and subsequent formation of water at the surfaces is an alternative route in the process whereby terrestrial planets accumulate water. Alternatively, surface adsorption could aid in the reaction of two hydrogen atoms to form gaseous H_2_. It is this latter process that has been investigated in the most depth in previous studies. Experimental work by Vidali *et al.* [14–16] showed that, while H_2_ formation in the gas-phase interstellar medium is unlikely, the process could be catalysed by amorphous and disordered surfaces with the formula (Mg_0.5_Fe_0.5_)_2_SiO_4_. Also, the possible activity of small clusters (less than 15 Ådiameter) with structures based on pyroxene has been considered using computational methods [17].

The interactions of hydrogen with the crystalline olivine {010} surface have previously been studied experimentally, finding that H_2_ formation is thermally activated with a barrier dependent on the surface structure [18]. Theoretical work on this surface, using a quantum mechanics/molecular mechanics (QM/MM) methodology, supports these findings and suggests the possibility of both physisorption and chemisorption on the surface depending on the site considered [19,20]. A number of kinetic Monte Carlo studies have been performed in the past, covering a wide range of temperature and pressure conditions. This work highlights the complexity of the process, involving adsorption, surface diffusion and desorption of several species, as well as the possibility of physisorption–chemisorption transitions as mentioned previously [21,22].

Olivine minerals are silicate structures of the general formula M_2_SiO_4_, where M is magnesium and/or iron in varying proportion. The pure magnesium end-member forsterite (Mg_2_SiO_4_) and pure iron end-member fayalite (Fe_2_SiO_4_) thus lie at opposite ends of the olivine solid solution [23]. Previous studies have considered the thermodynamics and elastic properties of forsterite–fayalite mixtures [24], but in this work we will focus on the two pure end-member species, which will enable us to compare their surface structures and clearly distinguish their tendency towards the adsorption of hydrogen.

The {010} surface is the primary cleavage plane of olivine, and therefore has been studied in greatest detail in previous studies, at least for forsterite [10,25–27]. In this work, we will therefore also focus on the {010} surfaces in a comparison between the two end-member minerals, as results on fayalite have not been reported in the literature. We will consider first any surface restructuring or relaxation of the clean faces, before considering the adsorption of hydrogen. It has been suggested in previous computational studies [26,27] that the stability of the {010} surface of forsterite prohibits the strong chemisorption of water to this low-index face, but one can imagine that an olivine grain in an astrochemical environment (where low temperatures prevent rapid desorption once a species has attached to the surface) may still be able to retain adsorbed hydrogen long enough to catalyse surface reactions. As such, we have selected the {010} surface of both forsterite and fayalite to consider the adsorption of atomic hydrogen, which will enable us to compare with previous work on forsterite, but to extend this insight into a quantitative comparison with the fayalite surface.

Following on from calculations of the interaction of the defect-free {010} surfaces with hydrogen atoms, we have also considered the case where a metal atom vacancy in the surface layer is compensated by the adsorption of two hydrogen atoms on the closest surface oxygen sites. Previous work has considered defects of this type, a simile of the hydrogarnet defect [28], but commonly only for bulk minerals [27–30]. In an astrochemical environment, where minerals such as olivines may be built up atom-by-atom over long time scales or through low-impact collisions, such surface defects could be formed relatively easily.

## 2. Methodology

The calculations in this work were carried out using a plane-wave plus pseudopotential method based on the density functional theory (DFT), as implemented in the Vienna Ab-initio Simulation Package (VASP) [31–34], with projected augmented wave functions within the generalized gradient approximation (GGA) level of theory, using the Perdew–Burke–Ernzerhof exchange-correlation functional [35–37].

Unit cell parameters for forsterite and fayalite were relaxed under constant-volume conditions in order to avoid errors relating to the Pulay stress [38]. Energies obtained for a range of cell volume scaling constants were fitted to the Murnaghan equation of state [39], giving minimum energy unit cell structures for which the ion positions were subsequently relaxed. Bulk calculations made use of a 400 eV plane-wave cutoff energy and an 8×4×8 Monkhorst–Pack *k*-point sampling grid. The same cutoff, but with 3×1×3 or 3×3×1 *k*-point grids, was used in surface calculations. All geometry relaxations were performed with a convergence criterion of 0.01 eV Å^−1^. Spin polarization was enabled throughout all calculations, while dipole corrections were avoided in order to more accurately represent the true conditions.

Surface structures were generated using the program METADISE [40] and compared with earlier work on forsterite using interatomic potentials, which suggested that a surface region of over 10 Åwas required to obtain a converged surface structure. Slabs were therefore created with a total thickness of 20 Å, consisting of around 5 Åin the lower section, which was frozen in the optimized bulk configuration, plus 15 Å, which was allowed to relax. The vacuum gap above the slab was set to around 15 Å. These calculations proved to be too computationally costly for the adsorption studies, and as the optimized surfaces were found not to undergo significant restructuring, the slabs were therefore reduced to around 8 Åthickness, with a single layer frozen to maintain bulk-like geometry. Once again, a vacuum gap of 15 Å was used, which was sufficient to accommodate adsorbate atoms at a range of elevations above the surface.

## 3. Results and discussion

**Figure 1.**
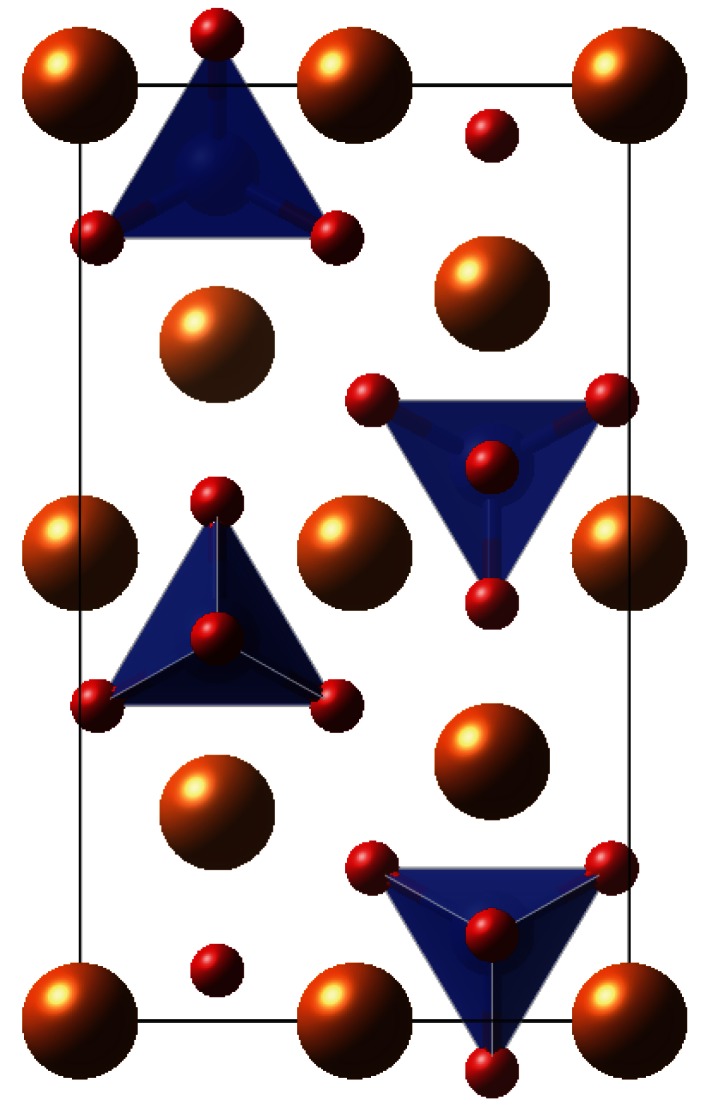
Bulk unit cell geometry of olivine material such as forsterite or fayalite. O, red; Mg or Fe, gold; Si, blue; SiO_4_ units shown as tetrahedra.

Olivine has *Pbnm* symmetry and a structure composed of a distorted hexagonal close-packed oxygen lattice, with half of the O_h_ voids occupied by metal cations and one-eighth of the T_d_ sites filled by Si atoms (figure 1). The optimized unit cell parameters for the minerals are presented in table 1, and these bulk structures were used to construct 1×1 surface cells for the {010} surface of each material. The surface structure of forsterite, obtained using METADISE, was evaluated against previous work for consistency.

**Table 1.** Unit cell parameters obtained for forsterite and fayalite after unit cell optimization in VASP. Values in brackets are experimental data.

	*a* (Å)	*b* (Å)	*c* (Å)
forsterite	4.810 (4.795)	10.268 (10.356)	6.029 (6.060)
fayalite	4.606 (4.773)	10.009 (10.252)	5.818 (6.026)

### (a) Surface structures

**Figure 2.**
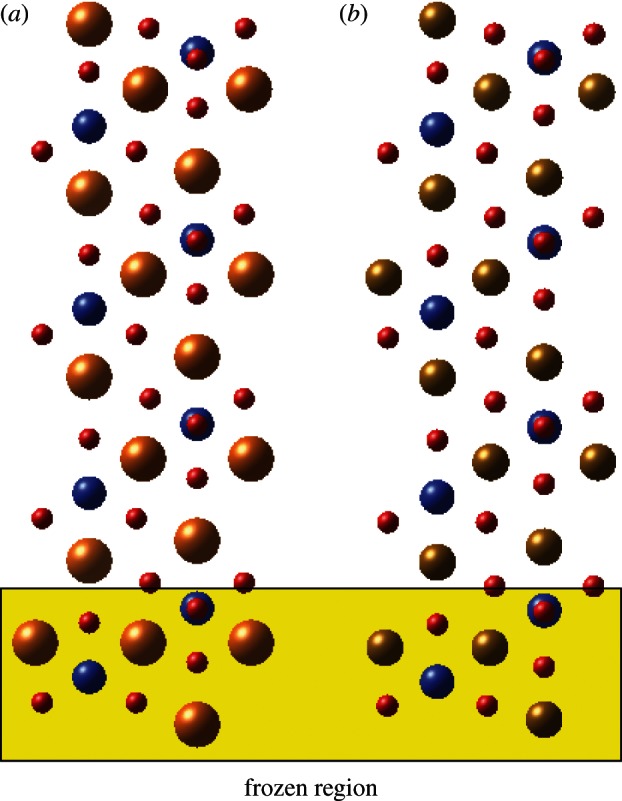
Surface slabs for the {010} terminations of (*a*) forsterite and (*b*) fayalite. O, red; Mg or Fe, gold; Si, blue; the frozen region, approximately 5 Å thick, is highlighted in yellow.

Large slabs were constructed for the {010} surfaces of forsterite and fayalite in order to study the extent of relaxation, shown in figure 2. It is clear on inspection that no significant structural differences occur upon relaxation of the surfaces of the two materials, most clearly seen by comparing the relaxed surface at the top with the frozen region at the bottom of each slab in figure 2. The only significant structural change is the downward shift of the surface metal cation by 0.63 Åfor forsterite and 0.48 Å for fayalite. This shift in the metal atom positions leads to a reduction in metal–oxygen distances of around 0.1–0.15 Å. The surface energies for the two surfaces, obtained from equation (3.1), were calculated at *γ*=1.26 J m^−2^ for forsterite and *γ*=1.04 J m^−2^ for fayalite:
3.1
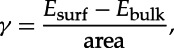
where *E*_surf_ is the total energy of the slab of material containing the surfaces, *E*_bulk_ is the energy of a portion of bulk material containing the same number of stoichiometric (Mg,Fe)SiO_4_ units and area is the surface area. The lower value of the surface energy of the fayalite {010} surface, compared with the same plane in forsterite, could indicate that the fayalite surface may be more stable and should be formed more easily, although it is difficult to make a direct comparison between the two compositions. The surface energy of the forsterite {010} surface is very similar to the value of 1.28 J m^−2^ reported in the literature for interatomic potential simulations of the same plane [10,26], suggesting that both methods have captured adequately the essential structural description of the materials.

### (b) Hydrogen adsorption

**Figure 3.**
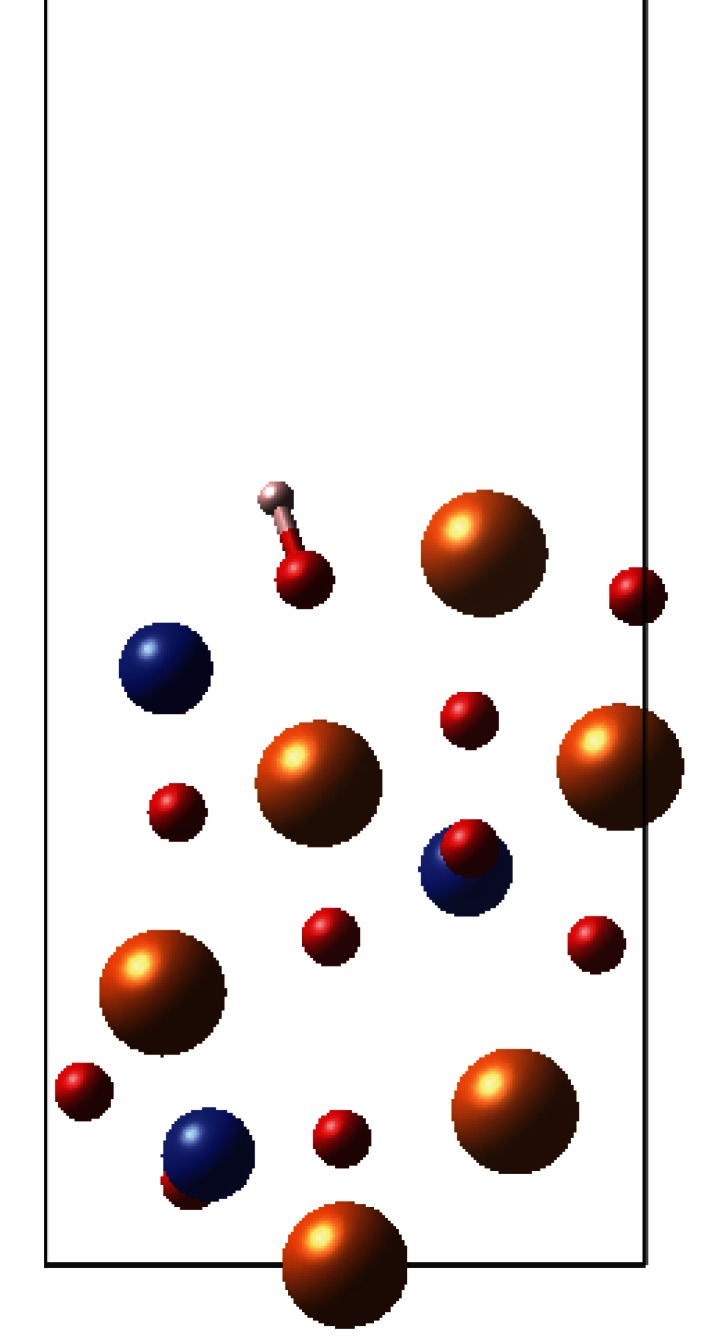
Small surface slab used for adsorbate calculations.

As mentioned earlier, a smaller slab was used in order to study the hydrogen adsorption processes, as no evidence was found of significant long-range relaxation of the surface slabs. An example slab, with adsorbed hydrogen, is shown in figure 3, where the lowest layer of atoms was fixed at the optimized bulk positions, while all other atoms were allowed to relax.

The energy of adsorption for a single hydrogen atom on the olivine surface was defined as follows:
3.2
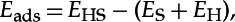
where the energy of the hydrogen+surface structure (*E*_HS_) is obtained by performing a structural optimization after the H atom has been placed at a sensible distance above the surface site under consideration. The energy of the surface slab (*E*_S_) and hydrogen atom (*E*_H_) may be obtained separately, or alternatively as a single term from a second hydrogen+surface calculation, but in this case with a large separation between the surface and incoming hydrogen atom (greater than 5 Å). In this study, we have opted for the latter scenario in order to avoid complications due to the need to maintain a consistent basis set, which could lead to uncertainties in the calculated results.

**Figure 4.**
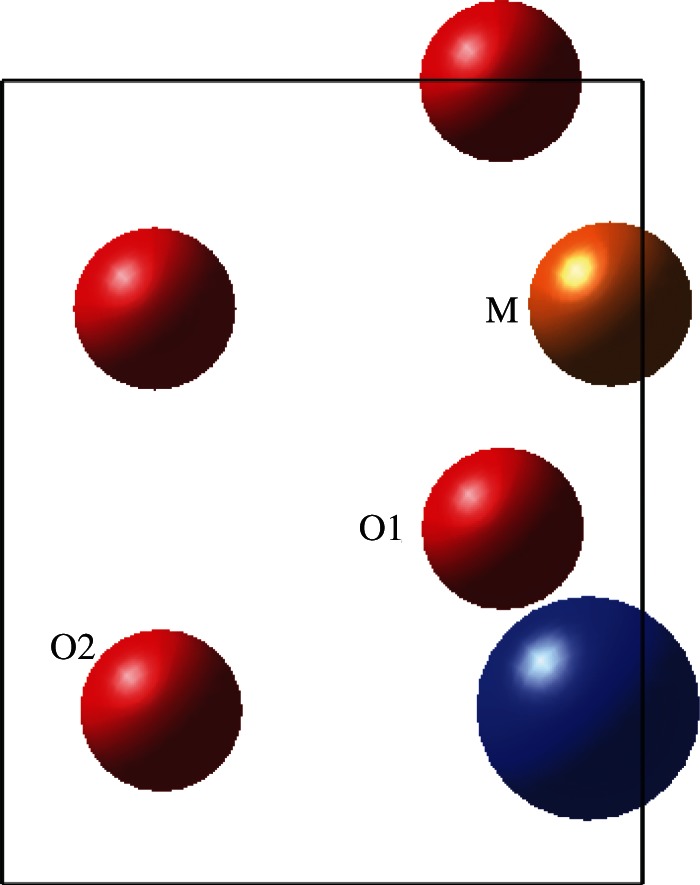
Surface adsorption sites. Plan view of the {010} surfaces, where for clarity only atoms exposed at the surface are shown here. Mg, Fe, gold; O, red; Si, blue.

Three surface sites were initially considered for adsorption of hydrogen, labelled as M, O1 and O2 in figure 4. Site O1 appears twice on the surface structure due to symmetry of the olivine structure; O1 is the oxygen atom that is most elevated out of the surface plane and therefore most accessible to incoming hydrogen atoms. As such, we would expect O1 to be more reactive towards adsorbates, but we have also considered O2 for comparison.

In addition to calculating the energy of adsorption of hydrogen on the different surface sites, we have also determined the charge on the H atoms during the adsorption process. The total charge output by VASP refers to the electron density situated within the Wigner–Seitz radius for each atom, which is generally defined as part of the pseudopotential specification. Owing to the recognized problem of over-delocalization of electrons in GGA–DFT due to the self-interaction error, values of total charge obtained in this way give only an approximate view of the true charge on the atoms in the system. Nevertheless, the data should be sufficient to provide insight into the change in charge occurring as a result of adsorption. In all cases where a hydrogen atom was placed at a large distance from the surface (including calculations on an isolated H atom), the charge assigned to hydrogen was found to be around 0.381 e. This appears to be reasonable, as the Wigner–Seitz radius for hydrogen is small, allowing for the rest of the electron density to lie further away. Charges on the hydrogen atoms will be expressed as *Δq*, representing the difference between the hydrogen charge in the adsorbed system and this reference value of 0.381 e.

The adsorption energies for hydrogen atoms at each of the {010} surface sites for both forsterite and fayalite are listed in table 2. We can see that, in forsterite, energetically favourable adsorption of hydrogen was observed for the M and O1 sites, but not for O2 site; whereas, in fayalite, adsorption was energetically possible on all three sites. The reason for this difference is not clear, although it may be a geometric effect, as iron is a smaller cation than magnesium and may therefore shield the O2 site less effectively.

**Table 2.** Adsorption energies for a single hydrogen atom on three surface sites of forsterite and fayalite. Values in electron-volts, with negative values corresponding to favourable interactions.

	O1	O2	M
forsterite	−0.81	+0.06	−0.45
fayalite	−2.82	−2.24	−1.07

As well as considering the optimum hydrogen adsorption positions on the surfaces, we have attempted to calculate approximate activation barriers for the adsorption process. This calculation involved a simple constrained geometry search by shifting the H atom vertically away from the optimized site for a number of steps. This process assumes that the route to adsorption is a ballistic transportation of the hydrogen from vacuum directly downwards onto the surface site. Whereas this is not necessarily the lowest-energy pathway, we consider that it provides a reasonable model for the adsorption processes in an environment where the density of gaseous atoms and molecules is extremely low.

**Figure 5.**
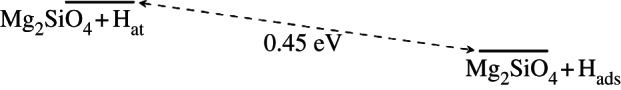
Representation of the hydrogen adsorption process from vacuum onto a surface Mg site in the forsterite {010} surface.

**Figure 6.**
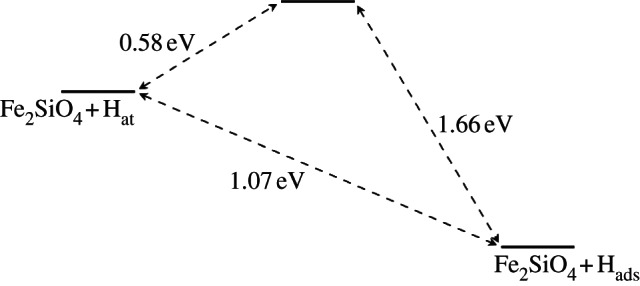
Representation of the hydrogen adsorption process onto a surface Fe site in the {010} surface of fayalite, showing an apparent energy barrier of 0.58 eV.

On the forsterite M site, hydrogen was found to adsorb with a fairly long Mg–H distance of 1.93 Å, releasing an energy of adsorption (*E*_ads_) of 0.45 eV without apparent energy barrier to the approach of the hydrogen. The change in hydrogen charge *Δq* was found to be very small, with only an extra 0.08 e located close to the H atom (a 20% increase). For fayalite, the situation differed significantly. The optimum position for the H atom was found to be at a distance of 1.51 Åfrom the Fe site, the energy of adsorption was increased to 1.07 eV, and *Δq* was substantially higher at 0.154 e. In the fayalite case, however, a barrier of 0.584 eV was found for the approach of the hydrogen to the surface, at a distance of around 2.54 Å. Figures 5 and 6 summarize the energetics of the hydrogen approach to the surface cations. The small charge density shift from the slab onto the hydrogen atoms could indicate the formation of hydride ions, which may allow for the formation of H_2_ gas if this species subsequently interacts with a proton.

We next considered the adsorption of a hydrogen atom at the O1 sites of forsterite and fayalite. Here, the situation observed for the interaction with cation sites was reversed—fayalite exhibited no barrier to H adsorption, whereas forsterite displayed an apparent energy barrier of 0.15 eV, with the adsorption directly downwards onto its minimum energy position being disfavoured at all heights, suggesting that adsorption directly above the O1 site followed by rearrangement once the bond is formed is preferred. Although small, this barrier could still significantly hinder the adsorption of hydrogen at this site in a low-pressure/temperature environment, such as that found in interstellar space. In both cases, adsorption was stronger than on the corresponding metal sites, with values for *E*_ads_ of 0.81 and 2.82 eV for forsterite and fayalite, respectively, and calculated O–H distances of 0.98 Åfor each mineral.

The hydrogen adsorption process at the O1 site on fayalite was the energetically most favourable interaction found in this study, and the results suggest that O–H bonds are formed readily whenever a free hydrogen atom encounters this adsorption site. Furthermore, such bonds are unlikely to break again without significant energy input, for example, at very high temperatures. In all cases of OH bond formation, no significant electron localization was observed, suggesting that the extra electron from the atomic hydrogen was delocalized over the adjacent atoms.

**Figure 7.**
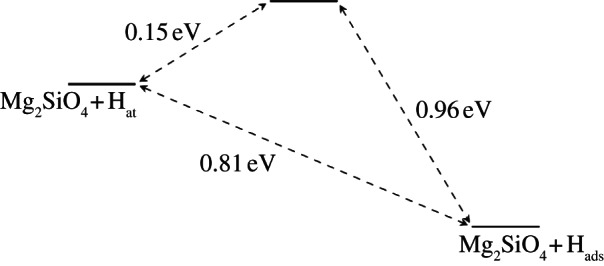
Schematic for the adsorption process onto the O1 site in the forsterite {010} surface.

**Figure 8.**
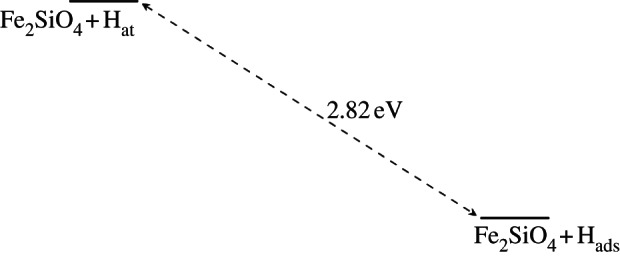
Schematic for the adsorption process onto the O1 site in the fayalite {010} surface.

A *Δq* of 0.33 e on the hydrogen was observed for both materials (an 86% increase) However, given the O–H distance and interaction strengths, this increase appears to be the result of O–H bond formation, leading to the hydrogen being located within the region of increased electron density surrounding the oxygen, rather than the creation of a hydride ion. Effectively, the extra charge on the hydrogen ‘belongs’ to the oxygen, and the proximity of the atoms simply leads to miscalculation owing to the simplicity of the process used to quantify charge density here. Although the charge density data for this site initially appear to suggest a similar outcome to adsorption on the M site, the substantial decrease in surface–hydrogen distances leads us to the conclusion that the behaviour observed on the O1 site corresponds to bond formation, while adsorption on the M site is more likely to involve charge transfer. Figures 7 and 8 summarize the two processes.

**Figure 9.**
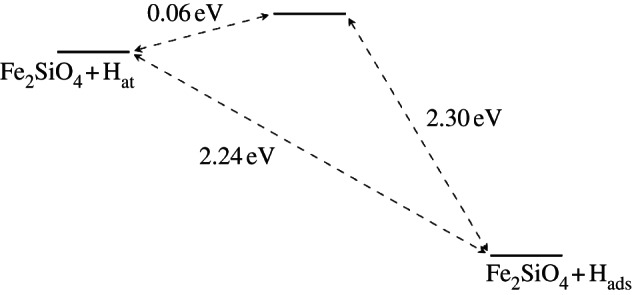
Schematic for the adsorption process onto the O2 site in the fayalite {010} surface.

Finally, we consider the interaction of hydrogen with the O2 site on the olivine surfaces. As mentioned earlier, no significant interaction was detected for forsterite, but in fayalite this O–H interaction was calculated to be strong; an *E*_ads_ of 2.24 eV was calculated at an adsorption distance of around 1.25 Å, along with a *Δq* of 0.32 e (83% increase in electron density in the vicinity of the hydrogen). Similar to the O1 site, H is chemisorbed to form a strong O–H bond on fayalite, although this time a small barrier of 0.06 eV was observed (figure 9) at around 1.63 Åelevation. The presence of this energy barrier compared with the barrier-free adsorption at the O1 site suggests that adsorption at the O1 site would be more likely. However, this argument does not include consideration of surface migration after initial adsorption. Even so, the O1 site forms a strong O–H bond, stronger than O2–H in fact, indicating that it is likely that hydrogen would only adsorb readily at the O2 sites once the nearest O1 sites are saturated.

### (c) Hydrogen adsorption at surface defects

Work on the defect-free olivine surfaces was complemented by the investigation of hydrogen adsorption as a possible compensation method for the presence of metal cation vacancies. In each case, the metal atom (Mg or Fe) was removed and hydrogens placed on each of the two O1 sites adjacent to the vacancy. Geometry optimization was then carried out to obtain realistic O–H bonding geometries. The defect formation energy for the removal of a metal species from the surface was defined as follows:
3.3
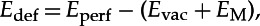
where *E*_M_ is the energy of a metal atom in isolation. In an astrochemical environment, a gas-phase metal atom provides a more valid point of comparison than bulk metal, especially as we are considering the possibility of a defect site that could potentially be filled at a later point by collision with a free metal atom. This approach also means that both the vacant species and the slab are uncharged, and no electronic compensation is necessary. *E*_perf_ and *E*_vac_ correspond to the energies of the defect-free and defective surfaces, respectively.

**Figure 10.**
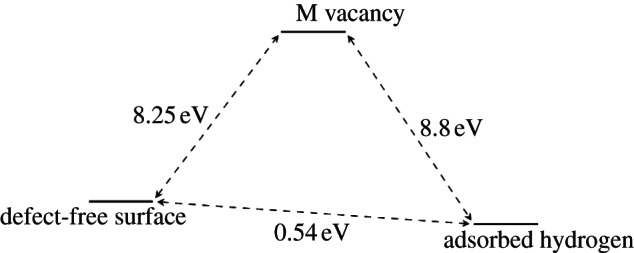
Energy diagram of the various states of the defect-free and Mg-vacant forsterite surface.

**Figure 11.**
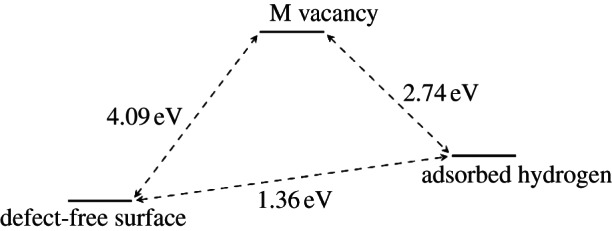
Energy diagram of the various states of the defect-free and Fe-vacant fayalite surface.

Figures 10 and 11 show the schematic energy diagrams for the adsorption of hydrogen at the metal vacancy sites, also including the energy of removing the metal atoms to create the olivine surfaces with Mg and Fe vacancies. For forsterite (figure 10), the surface structure with two adsorbed hydrogen atoms compensating for the loss of an Mg atom is calculated to be slightly more favourable than the unmodified surface. Furthermore, if we assume a pre-existing Mg vacancy in the surface, due to the natural mineral growth process, it is clear that the energy released upon adsorption of a hydrogen atom at the O1 site adjoining the vacancy (*E*_*ads*_=4.4 eV/H atom) is considerable and the hydroxy groups thus formed are unlikely to become deprotonated, even at very high temperatures. The process is significantly different for the fayalite surface (figure 11). Here, the replacement of Fe by two hydrogens is overall an energetically unfavourable process, but whereas in forsterite the defect formation energy of the Mg vacancy is extremely high at around 8.25 eV, this is more than compensated by the addition of two hydrogen atoms, giving an overall energy for the exchange of Mg by two H atoms of 0.54 eV. For fayalite, however, the defect formation energy is significantly lower at 4.09 eV for the formation of an Fe surface vacancy. However, the adsorption of hydrogen does not, in this case, lead to a system that is more stable than the original defect-free surface. The adsorption of a hydrogen atom at the O1 site near the Fe vacancy to form a hydroxy ion only releases *E*_ads_=1.37 eV/H atom, whereas the hydrogen-compensated fayalite surface is less stable by about 1.36 eV than the clean {010} surface.

**Figure 12.**
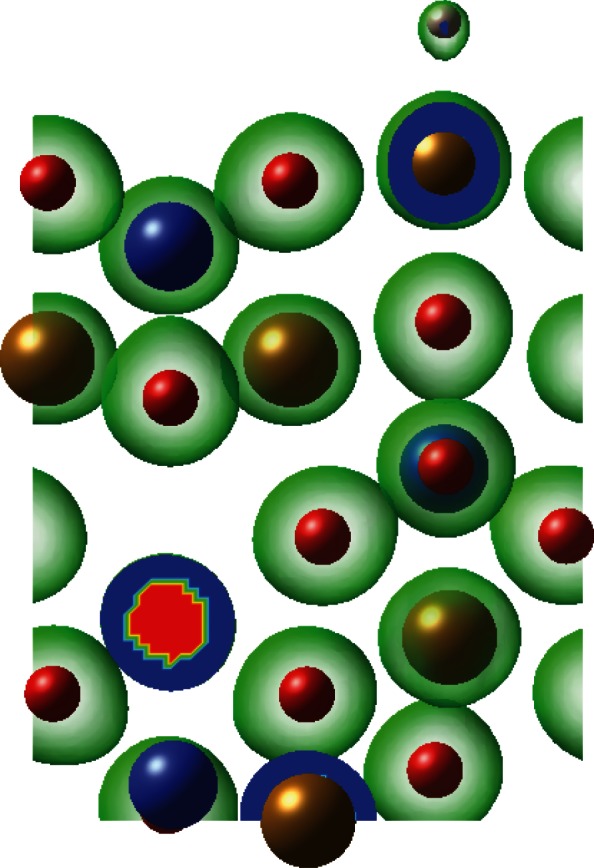
Example charge density plot for a physisorbed hydrogen over the M site in fayalite.

**Figure 13.**
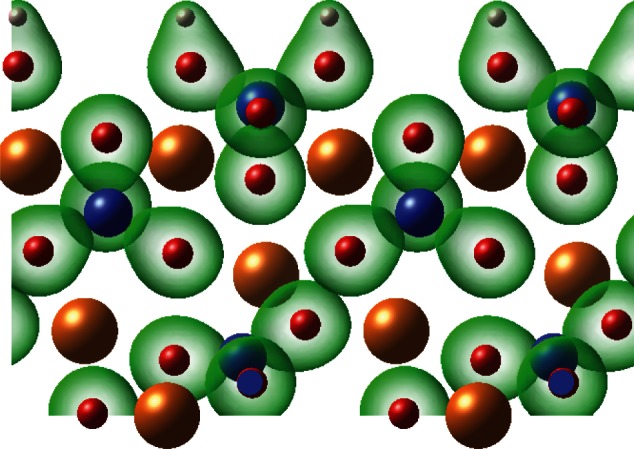
Charge density plot showing O–H bond formation over the surface of forsterite with an Mg atom vacancy. Also clearly visible are the deformations of charge density around oxygen towards Si ions.

Figures 12 and 13 show examples of charge density iso-surface plots for surface–adsorbate interactions, where the same iso-surface value was used in each case. We can clearly see the difference between a loosely bound H atom (possibly a hydride ion) over the fayalite M site (figure 12) and O–H covalent bonds in the forsterite vacancy substitution case (figure 13).

## 4. Conclusions

We have modelled the bulk and {010} surface of the olivine material, and compared the differences between the Mg and Fe end-members of the material, which showed that the effect of the cation type on the surface structure is insignificant.

Next, we have considered a range of hydrogen adsorption scenarios on both the defect-free {010} surfaces of forsterite and fayalite, as well as hydrogen adsorption near cation vacancies in the surface. Adsorption of a single hydrogen atom appears to be favoured on the fayalite surface compared with the forsterite surface at both surface oxygen sites and the metal site, although in some cases an energy barrier is identified in the adsorption process. However, only a vertical adsorption from vacuum onto the surface site is considered, whereas a less direct and more lateral adsorption pathway may lower the calculated energy barriers, which should therefore be seen as maximum energy barriers to adsorption of hydrogen from the vacuum. We speculate that the stronger interaction of the H atom with the fayalite surface may be due to the presence of the redox-active Fe species in fayalite, which will facilitate redistribution of the electron density. Although Fe(I) species are known, especially when complexed by ligands, as shown, for example, by electron paramagnetic resonance spectroscopy [41], this effect is particularly notable when hydrogen is adsorbed at the metal site, where the increase in electron density on the hydrogen is significantly higher on the fayalite surface than on the forsterite surface, indicating that the formation of a hydride ion is enhanced at the Fe(II) site, which can of course be fairly easily oxidized to Fe(III). As protons are formed upon hydrogen adsorption at the surface oxygen sites, the formation and presence of both hydride ions and protons in the form of hydroxy groups on a single surface indicates the possibility of surface-activated formation of dihydrogen species as well as water molecules.

Calculations of hydrogen adsorption at a metal vacancy on the {010} surfaces suggest that the hydrogen-compensated system is energetically preferred over the defect-free {010} surface of forsterite (relative to an isolated Mg in the vacuum phase), while for fayalite the situation is reversed and adsorption of two hydrogen atoms at the neighbouring oxygen sites compensates only partially for the energetic cost of creating the metal vacancy.

Findings for the lowest-energy structures of hydrogen over the forsterite surface compare well with the embedded cluster QM/MM work of Goumans *et al*. [19], where physisorption over the Mg site (approx. 1.8 ÅMg–H separation) and chemisorption over an O site (0.97 ÅO–H bond) were deemed to be the most probable interactions with the unmodified {010} surface. The fact that our results give essentially the same conclusions using a significantly different model gives us added confidence in the accuracy of the calculations involving fayalite and the defective surfaces.

Further work will extend this study to include hydrogen adsorption at alternative olivine surfaces to compare their reactivity to that of the dominant {010} surfaces. Other avenues to explore include the use of nudged elastic band techniques to study surface diffusion of protons and hydrides and the formation of molecular hydrogen and/or water at the surface, and the modelling of solid solutions of the Mg,Fe olivines to study the effect of the presence of both cations at the surface on the hydrogen adsorption processes. Finally, we will also investigate the use of embedded cluster calculations to increase the surface area of the relevant surfaces, firstly to evaluate possible sources of error arising from the presence of periodic image interaction, and secondly to obtain surface hydrogen coverages that are more realistic considering the low concentration of hydrogen in the interstellar medium.
